# TrkB Truncated Isoform Receptors as Transducers and Determinants of BDNF Functions

**DOI:** 10.3389/fnins.2022.847572

**Published:** 2022-03-07

**Authors:** Lino Tessarollo, Sudhirkumar Yanpallewar

**Affiliations:** Neural Development Section, Mouse Cancer Genetics Program, Center for Cancer Research, National Cancer Institute, Frederick, MD, United States

**Keywords:** TrkB.T1, BDNF, TrkB truncated, splicing, neurodegeneration

## Abstract

Brain-derived neurotrophic factor (BDNF) belongs to the neurotrophin family of secreted growth factors and binds with high affinity to the TrkB tyrosine kinase receptors. BDNF is a critical player in the development of the central (CNS) and peripheral (PNS) nervous system of vertebrates and its strong pro-survival function on neurons has attracted great interest as a potential therapeutic target for the management of neurodegenerative disorders such as Amyotrophic Lateral Sclerosis (ALS), Huntington, Parkinson’s and Alzheimer’s disease. The TrkB gene, in addition to the full-length receptor, encodes a number of isoforms, including some lacking the catalytic tyrosine kinase domain. Importantly, one of these truncated isoforms, namely TrkB.T1, is the most widely expressed TrkB receptor in the adult suggesting an important role in the regulation of BDNF signaling. Although some progress has been made, the mechanism of TrkB.T1 function is still largely unknown. Here we critically review the current knowledge on TrkB.T1 distribution and functions that may be helpful to our understanding of how it regulates and participates in BDNF signaling in normal physiological and pathological conditions.

## Introduction

In mammals, the neurotrophin family is comprised of four members: nerve growth factor (NGF), brain-derived neurotrophic factor (BDNF), neurotrophin-3 (NT3), and neurotrophin-4/5 (NT4/5). Two types of receptors mediate their actions, the Trk family of tyrosine kinase receptors and the p75 NGF receptor, a member of the tumor necrosis factor receptor superfamily. Binding experiments with cell lines were used to determine the ligand-receptor relationship between neurotrophins and their receptors. NGF binds to TrkA, whereas BDNF and NT4/5 bind to TrkB. NT-3 signals mainly through TrkC but can also bind with lower affinity to TrkA and TrkB (Bothwell, 1995; Chao and Hempstead, 1995; Friedman and Greene, 1999; Kaplan and Miller, 2000; Huang and Reichardt, 2003). The phenotypes observed in mice with targeted mutations in neurotrophins and their Trk receptors have demonstrated the specificity of these interactions, particularly in the developing peripheral nervous system (PNS; Snider, 1994; Tessarollo, 1998). Trk receptors, upon ligand binding, activate well-known intracellular signaling cascades such as the Ras/MAPK pathway, phosphoinositide 3 kinase and phospholipase Cγ (PLCγ) pathways. In the early experiments of gene targeting of the Trk genes in mice, the mutations were directed toward the tyrosine kinase domain region of the full-length receptors because of the established biological activities of these isoforms. However, Trk genes by alternative splicing can produce a wide array of other isoforms. For many of these isoforms the pattern of expression, the conservation among species, and their physiological abundance has not yet been elucidated (Tsoulfas et al., 1993; Luberg et al., 2010, 2015). However, the TrkB and TrkC genes, by alternative splicing generate isoforms that lack tyrosine kinase activity, are conserved among species and are expressed at high levels (Klein et al., 1990; Middlemas et al., 1991; Tsoulfas et al., 1993; Valenzuela et al., 1993; Garner and Large, 1994; Luberg et al., 2010, 2015). Though they were first discovered about 30 years ago, only recently we have begun to learn about the function of these types of receptors. This was in part due to their lack of tyrosine kinase activity, unclear signaling and no obvious pro-survival effect on neurons. The lack of pro-survival functions on different neuronal populations was confirmed *in vivo* with the second generation of mouse models targeting all Trk genes isoforms showing only minimal phenotypic differences compared to the initial mouse models targeting the kinase domain (Klein et al., 1993, 1994; Smeyne et al., 1994; Tessarollo et al., 1997; Liebl et al., 2000; Luikart et al., 2005). Thus, for a long time, the main functions attributed to these truncated receptor isoforms were of acting as dominant-negative inhibitors of the full-length receptors or limiting ligand availability (Biffo et al., 1995; Eide et al., 1996; Palko et al., 1999). In 2003 (Rose et al., 2003), the report that TrkB.T1 can signal independently by inducing calcium release from the intracellular stores, and in 2006, the generation of a new mouse model targeting specifically the exon encoding the TrkB.T1 isoform without affecting the spatio-temporal pattern of expression of TrkB full-length (TrkB.FL; Dorsey et al., 2006) started a new research effort into the functional role of these enigmatic receptors. As outlined below this research has led to new exciting and unanticipated functions for TrkB.T1 in mammalian physiology. Truncated TrkC isoforms signal by binding the scaffold protein Tamalin which in turn activates the Rac1 GTPase through the adenosine diphosphate-ribosylation factor 6 (Esteban et al., 2006). Moreover, it has also been shown that TrkC.T1 can lead to neural differentiation in collaboration with p75NTR (Hapner et al., 1998). However, the generation of a suitable mouse model to study TrkC truncated receptors *in vivo* has been hampered by the fact that targeting of the exons encoding specifically the truncated TrkC.T1 (also known as TrkC.NC2) leads to dysregulation of the TrkC.FL receptor expression (Bai et al., 2010). Therefore, the lack of a proper mouse model has limited our ability to study the precise function of TrkC.T1 *in vivo*. In this review, we focus on the current knowledge on truncated TrkB.T1, the most studied truncated Trk receptor to date. We discuss the many *in vivo* functions that have been uncovered so far and its relevance in the pathophysiology of neuronal disorders in both animal models and human diseases.

## Alternative Splicing as a Mechanism to Diversify Brain-Derived Neurotrophic Factor/TrkB Signaling

The finding that the mouse and human genome contain fewer genes than previously thought have underlined the importance of alternative splicing as a mechanism to build more complex organisms (Lander et al., 2001; Venter et al., 2001; Waterston et al., 2002a,b). Importantly, alternative splicing in mammals (Clancy, 2008; Donaldson and Beazley-Long, 2016) is particularly prominent and highly conserved in the brain (Raj and Blencowe, 2015). Functionally, alternative splicing can result in protein isoforms that are inactive or exhibit similar, different, or even opposing actions. Moreover, alternatively spliced transcripts and their protein isoform products show dynamic changes in expression and function that are dependent on cell, tissue, age, and context (physiological vs. pathological). BDNF and its receptor TrkB are good examples of genes whose function is tightly associated with alternative splicing. The BDNF gene consists of at least 8 different promoters that can generate 18 separate transcripts. While all transcripts generate the same BDNF polypeptide, the presence of different promoters allows for the differential regulation of BDNF expression. For example, in the cortex, promoter IV-dependent transcription is responsible for activity-induced BDNF expression (Timmusk et al., 1993; Timmusk and Metsis, 1994; Tao et al., 1998; Aid et al., 2007; Hong et al., 2008). In addition, the different mRNA variants can be transported to differential subcellular locations for local translation of BDNF leading to selective morphological remodeling of dendrites (Baj et al., 2016). The human TrkB gene, spanning about 400 kbp, consists of 24 exons that through a complex pattern of alternative splicing can generate up to 30 TrkB isoforms (Stoilov et al., 2002; Luberg et al., 2010). However, despite the abundance of potential TrkB isoforms the most highly expressed isoforms in the mammalian brain are the TrkB.FL and TrkB.T1 receptors (Tomassoni-Ardori et al., 2019). These TrkB receptors are differentially expressed within the diverse areas of the nervous system in a spatial and temporal fashion (Stoilov et al., 2002; Luberg et al., 2010). Developmentally, TrkB.FL is predominantly expressed in the embryonic and early postnatal CNS whereas TrkB.T1 expression is very low in the embryo but increases gradually postnatally and peaks in adulthood (Escandon et al., 1994). At the cellular level, in the nervous system, expression of TrkB.FL is restricted to neurons whereas TrkB.T1 is expressed in both neurons and glia cells (Sommerfeld et al., 2000; Dorsey et al., 2006; Holt et al., 2019; Tomassoni-Ardori et al., 2019). Interestingly, outside the nervous system, TrkB.T1 appears to be the main isoform as it is expressed in the adult heart, kidney, lung, and pancreas (Stoilov et al., 2002; Fulgenzi et al., 2015, 2020).

## Regulation of TrkB.T1 Receptor Isoform Expression

**FIGURE 1:**
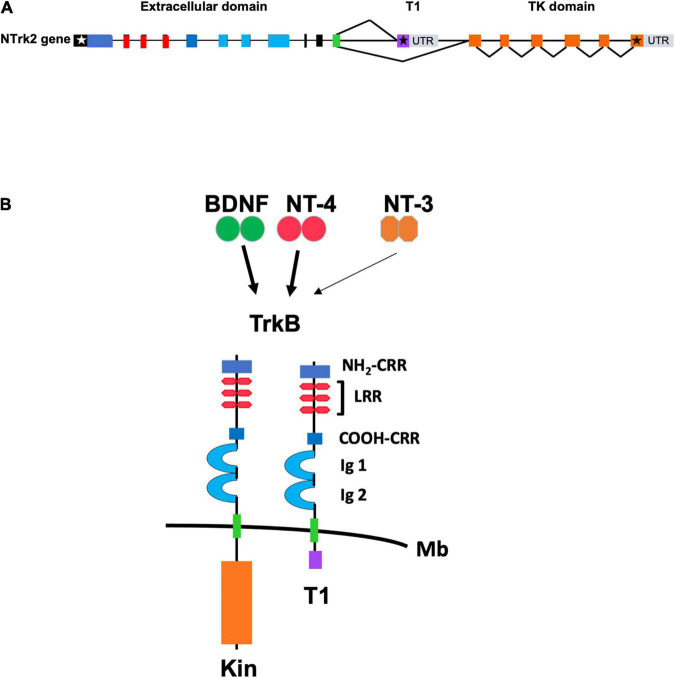
**(A)** Schematic representation of the genomic structure of the murine TrkB gene. Exons are shown as boxes and introns are shown as lines. White and black stars indicate the start and stop condons, respectively. Color of exons indicate the domains of the TrkB protein isoforms shown in panel **(B)**. Gray boxes indicate the 3′ untranslated region (UTR) of the TrkB transcripts. T1 and TK (Tyrosine Kinase) domain indicate the exons encoding, respectively, the TrkB.T1 and the TrkB.FL isoform that are produced by alternative splicing. **(B)** Schematic representation of the TrkB tyrosine kinase and TrkB.T1 isoform receptors. The extracellular TrkB protein domains include the amino (NH_2_-CRR) and carboxy (COOH-CRR) cysteine rich region; the leucine rich region (LRR) and the IG like, immunoglobulin like-domain (Ig1 and Ig2). The intracellular tyrosine kinase (Kin) and T1 domain are indicated below the cell membrane (Mb). BDNF and Neurotrophin-4 (NT-4) are the TrkB ligands binding with high affinity (solid arrow) whereas Neurotrophin-3 (NT-3) binds to the extracellular domain with lower affinity (thin arrow).

As mentioned above, the TrkB locus produces many different TrkB receptor isoforms by alternative splicing. However, very little is known about the mechanisms regulating their spatial and temporal expression. TrkB.T1 is the major isoform expressed in the adult mammalian brain and the best characterized among all known truncated isoforms. It includes the exons encoding the extracellular, transmembrane and the juxtamembrane domain that are common to the TrkB.FL receptor up to exon 15. After exon 15, instead of including exon 17 which is the first exon specific to the TrkB.FL isoform, the splicing machinery uses exon 16 which encodes a short intracellular 11 amino acid tail (FVLFHKIPLDG) that is unique to the TrkB.T1 isoform (Figure 1). This sequence lacks obvious homology to any known protein motifs but is 100% conserved between rodents, human and chicken (Klein et al., 1990; Middlemas et al., 1991; Biffo et al., 1995; Luberg et al., 2010). In addition to the 11 aa tail, exon 16 includes a stop codon and has its own 3′UTR sequence with a number of polyadenylation sites. The mechanism underlying the coding of TrkB.T1 versus the TrkB.FL isoform by alternative splicing is unknown and deserves further study. So far, only mechanisms regulating the levels of expression of the different isoforms have been described. For example, TrkB.T1 downregulation in the cortex of suicide victims appears to result from combined epigenetic mechanisms, including methylation of the promoter and the 3′UTR DNA sequence, histone modifications, and microRNA binding (Ernst et al., 2009, 2011; Maussion et al., 2012). More recently, the RNA binding protein RbFox1 has been shown to increase the levels of TrkB.T1 in the hippocampus by direct binding to the TrkB.T1 mRNA. In turn, TrkB.T1 upregulation impairs BDNF-dependent LTP which can be rescued by genetically restoring TrkB.T1 levels strongly suggesting that TrkB.T1 regulates important brain functions (Tomassoni-Ardori et al., 2019).

## Mechanisms of TrkB.T1 Signaling

### Dominant-Negative Regulation of TrkB.FL Signaling

**FIGURE 2:**
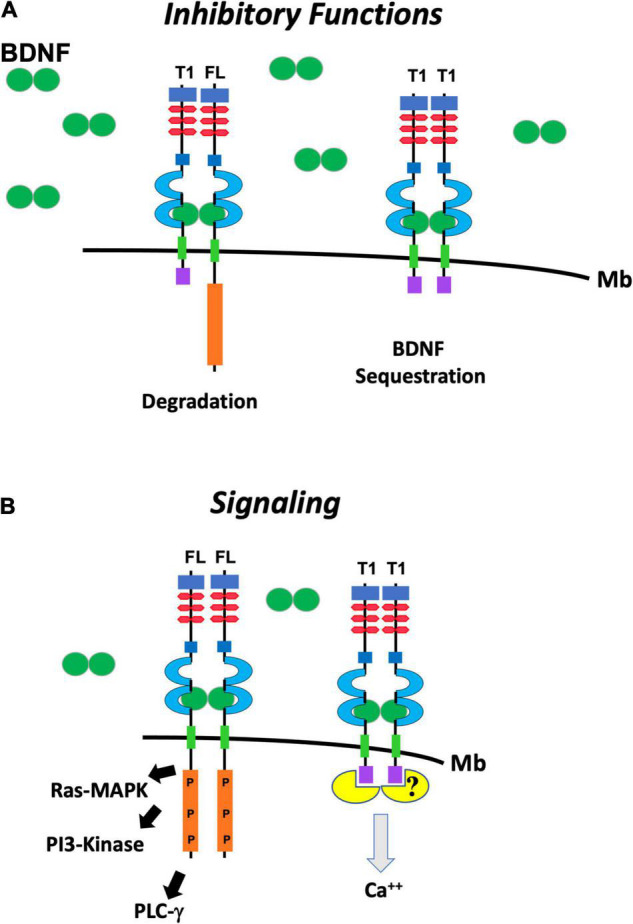
Schematic representation of the putative functions of TrkB.FL (FL) and TrkB.T1 (T1). **(A)** TrkB.T1 has inhibitory functions when it heterodimerizes with one isoform of TrkB.FL in response to BDNF (green ovals) binding and leading to internalization and degradation of the heterodimer (left). It also inhibits BDNF signaling by binding and sequestering it when it homodimerizes (right). **(B)** TrkB.FL (FL) homodimers in response to BDNF signal lead to auto-phosphorylation the kinase domain (P) and activation of the Ras/MAP Kinase (Ras-MAPK) pathway, phosphoinositide 3 kinase (PI3-Kinase) and phospholipase Cγ (PLCγ) pathways (left). TrkB.T1 homodimers can induce calcium (Ca++) release from the intracellular store through the binding and activation of a still unknown pathway (right).

The most studied mechanisms of TrkB.T1 signaling have been a dominant-negative role on TrkB.FL function and a BDNF scavenging action by limiting availability of the neurotrophin to activate TrkB.FL (Figure 2A). Small changes in TrkB.T1 levels can influence TrkB.FL activity because TrkB tyrosine kinase receptors can signal in response to extremely low concentrations (nano- to picomolar) of BDNF (Eide et al., 1996). The TrkB.T1 extracellular domain is identical to the TrkB.FL which allows it to engage BDNF with the same affinity of TrkB.FL and/or heterodimerize with a TrkB.FL monomer (Figures 1, 2; Biffo et al., 1995). Therefore, the formation of TrkB.T1 homodimers that sequester BDNF or TrkB.T1 heterodimers with the TrkB.FL isoform both impair BDNF signaling (Ohira et al., 2001). Several studies have supported these mechanisms. For example, co-expression of TrkB.T1 isoform with full-length receptor in xenopus oocytes prevents BDNF-induced activation of phospholipase C-γ pathway as measured by calcium efflux (Eide et al., 1996). Moreover, co-culture of the neuroblastoma cell line SY5Y expressing TrkB.FL, with NIH3T3 cells expressing TrkB.T1, inhibited neurite outgrowth in SY5Y cells under limiting concentration of BDNF (Fryer et al., 1997). Also, transfection of TrkB.T1 in a line of PC12 cells expressing TrkB.FL reduced its survival in the presence of BDNF and inhibited TrkB.FL autophosphorylation and kinase activity (Haapasalo et al., 2001), and pre-synaptic expression of TrkB.T1 in cultured hippocampal neurons prevented synaptic potentiation induced by BDNF (Li et al., 1998). Similarly, transfection of increasing amounts of TrkB.T1 in a cell line stably expressing TrkB.FL impairs BDNF-dependent TrkB.FL phosphorylation and signaling in a way that is directly proportional to TrkB.T1 levels. Conversely, TrkB.T1 knockout primary hippocampal neurons have higher basal, as well as BDNF-stimulated levels of p-TrkB.FL and p-ERK compared to control hippocampal neurons (Tomassoni-Ardori et al., 2019). These functions have also been supported by *in vivo* data as well showing that the level of expression of TrkB.T1 isoform has direct significant pathophysiological consequences. For example, in a mouse model with overexpression of TrkB.T1in postnatal cortical and hippocampal neurons, the induction of transient focal cerebral ischemia by middle cerebral artery occlusion causes significantly more neuronal damage as compared to controls. The increased damage occurs despite a BDNF mRNA upregulation in the peri-infarct region suggesting that increased TrkB.T1 limits BDNF function (Saarelainen et al., 2000). In addition, in the trisomy 16 (Ts16) mouse model there is increased apoptosis in the cortex and accelerated cell death of hippocampal neurons that cannot be rescued by administration of BDNF (Dorsey et al., 2006). This phenotype was mechanistically linked to increased TrkB.T1 expression since restoration of the physiological level of this isoform by gene targeting rescued Ts16 cortical cell and hippocampal neuronal death (Dorsey et al., 2006). Lastly, *in vivo* reduction of TrkB.FL signaling by removal of one BDNF allele could be partially rescued by TrkB.T1 deletion, which was revealed by an amelioration of the enhanced aggression and weight gain associated with BDNF haploinsufficiency (Carim-Todd et al., 2009).

### Regulation of Brain-Derived Neurotrophic Factor Signaling by TrkB.T1 Trafficking

TrkB.T1 also appears to have a distinct intracellular trafficking pattern compared to TrkB.FL. Binding of BDNF to TrkB.FL receptor results in rapid dimerization, transphosphorylation, and endocytosis. After internalization, the BDNF-TrkB complex is transported to various intracellular compartments which determines the type, strength, amplification, and duration of the downstream signaling cascades (Barford et al., 2017). Upon internalization, the receptors either go to the lysosomes for degradation or recycle back to the cell surface. TrkB.FL receptors predominantly get sorted to the degradative pathway resulting in downregulation of BDNF upon ligand-binding. In contrast, TrkB.T1 is predominantly recycled back (Sommerfeld et al., 2000; Chen et al., 2005) suggesting that the recycled TrkB.T1 can further sequester additional BDNF to regulate the duration of TrkB.FL mediated sustained activation of downstream MAP-kinase signaling (Huang et al., 2009). This function has been reported mainly for TrkB.T1 expressed in neurons (Sommerfeld et al., 2000; Chen et al., 2005). Additionally, TrkB.T1 expressed in astrocytes also appears to play a distinct role. TrkB.T1 in astrocytes isolated from rat hippocampi has been shown to mediate storage of endocytosed BDNF in a stable intracellular pool that can be used to release BDNF back into the extracellular environment over time (Alderson et al., 2000). Although the details regulating the sequestering and storage of BDNF mediated by TrkB.T1 endocytosis have not been fully elucidated, this represents another potential mechanism used for the fine-tuning of BDNF signaling.

### TrkB.T1 Independent Signaling

The first suggestion that TrkB.T1 could elicit signaling entirely independent of the full-length isoform was suggested by the Feinstein lab. Baxter et al. (1997) showed that TrkB.T1 is capable of mediating BDNF-induced signal transduction. More specifically, BDNF activation of TrkB.T1 increases the rate of acidic metabolite release from the cell, a common physiological consequence of many signaling pathways, and these changes occur with kinetics distinct from those mediated by TrkB.FL. Importantly, mutational analysis demonstrated that the specific intracellular domain of TrkB.T1 is essential for signaling (Baxter et al., 1997). Another key study showed that astrocytes that express TrkB.T1 respond to brief applications of BDNF by releasing calcium from intracellular stores. The finding that the calcium transients are insensitive to the tyrosine kinase blocker K-252a and persist in mutant mice lacking TrkB.FL strongly suggested a direct TrkB.T1 signaling role. While the study did not identify the downstream signaling mediators, pharmacologically, TrkB.T1-induced calcium release was found to be mediated by inositol-1,4,5-trisphosphate (Rose et al., 2003). Subsequently, it has been demonstrated that TrkB.T1 mediates BDNF-induced calcium release in cell types that exclusively express TrkB.T1 namely, cardiomyocytes and pancreatic β-cells (Fulgenzi et al., 2015, 2020). Lastly, there are a few isolated reports that have identified novel TrkB.T1 interactions and signaling mechanisms. It has been reported that the Rho GDP dissociation inhibitor 1 (GDI1), a GDP dissociation inhibitor of Rho small G-proteins, associates with TrkB.T1. This interaction appears to be constitutive but is disrupted by BDNF binding to TrkB.T1 (Ohira et al., 2005). Additionally, another 61 kDa protein has been reported to interact with TrkB.T1 but it has never been isolated (Kryl and Barker, 2000). In contrast to its role on calcium release, follow-up molecular studies on proteins directly interacting with TrkB.T1 have been lacking. Therefore, the characterization of the precise molecular pathways activated by TrkB.T1 is still largely unknown. It is possible that the small TrkB.T1 intracellular domain is unable to form stable interactions with proteins thus precluding their isolation and identification by mass-spectrometry.

### Physiological Roles of TrkB.T1

To prove a direct signaling function of TrkB.T1 *in vivo* has been challenging because the initial mouse model with a gene targeted mutation in the TrkB gene was generated by targeting the region encoding the tyrosine kinase domain (Klein et al., 1993). Although, this mouse model retained expression of the TrkB.T1 isoform because the TrkB.T1 encoding exon is upstream of the tyrosine kinase domain (see schematic in Figure 1A), it did not make it possible to determine a potential function of this truncated receptor due to the early postnatal lethality of the mutant. Other mouse models with either a complete deletion of all isoforms or selective inactivation of the kinase domain by a chemical genetic approach also did not allow determination of specific functions of TrkB.T1 because TrkB.FL exerts most BDNF pro-survival functions (Luikart et al., 2005; Johnson et al., 2008). To circumvent this problem, our laboratory targeted the exon specific to TrkB.T1 in mouse, generating a mouse model retaining the correct spatio-temporal pattern of expression of TrkB.FL. This mouse is viable but shows increased anxiety-like behavior accompanied by reduced length and complexity of dendritic arbors in basolateral amygdala (Carim-Todd et al., 2009). TrkB.T1 deletion also causes late onset cardiomyopathy because of defects in BDNF-induced calcium signaling and its role in cardiac contractility (Fulgenzi et al., 2015). Furthermore, loss of TrkB.T1 causes impaired glucose tolerance and insulin secretion due to its function in TrkB.T1-expressing pancreatic β-cells (Fulgenzi et al., 2020). In astrocytes, a cell type expressing high levels of TrkB.T1, its deletion causes immature morphology and reduced cellular volume as well as dysregulated expression of perisynaptic genes associated with mature astrocyte function (Holt et al., 2019). The lack of maturity by TrkB.T1 astrocytes may be the cause of slower *in vitro* migration in response to BDNF and reduced *in vivo* proliferation that has been associated with increased neuropathic pain and neurological dysfunction in TrkB.T1 KO mice following spinal cord injury (Matyas et al., 2017). The *in vivo* phenotypes caused by deletion of TrkB.T1 in cardiomyocytes, pancreatic β-cells and astrocytes, all cell types expressing this receptor isoform exclusively, provide definitive evidence of the independent signaling capability of TrkB.T1. One common feature of TrkB.T1 signaling in all these cell types is the activation of release of calcium from intracellular stores that, at least in glia cells, appears through a signaling pathway that involves an as yet unidentified “G protein” (Rose et al., 2003; Carim-Todd et al., 2009; Fulgenzi et al., 2015, 2020). It will be important to define whether the recorded calcium changes are indeed caused by TrkB.T1 association to a common G-protein or a specific G-protein unique to each cell type in which TrkB.T1 is expressed. Alternatively, TrkB.T1 could associate to some other adaptor or signaling proteins. Unbiased genome-wide targeting using the CRISPR/Cas9 system to inactivate the genes downstream of TrkB.T1 signaling may help identify all the components of this pathway/s. Lastly, the partial rescue by TrkB.T1 deletion of the enhanced aggression and weight gain associated with BDNF haploinsufficiency provides definitive *in vivo* evidence for a TrkB dominant/negative or BDNF sequestering role of TrkB.T1 (Carim-Todd et al., 2009). Taken together, over the last two decades the above studies have identified a variety of physiological roles for TrkB.T1 showing that it regulates a wide range of processes that extend beyond the nervous system.

## Role of TrkB.T1 in Disease

### Function in Cancer

Activation of the TrkB tyrosine kinase receptor has long been implicated in human tumorigenesis especially in the context of gain of function mutations associated with translocations and gene fusions (Eggert et al., 2001; Khotskaya et al., 2017). For these reasons, pan-Trk inhibitors of Trk tyrosine kinase receptors have been developed and were recently approved for cancer patients with NTRK fusion-positive solid tumors (Marcus et al., 2021). Whether TrkB.T1 plays a role in cancer biology has been less clear, mostly because of the lack of evidence of activation of specific pathways involved in cancer development and/or progression. Some studies, however, have reported a possible role for TrkB.T1 in tumorigenesis. Specifically, it has been shown that TrkB.T1 overexpression induces liver metastasis of pancreatic cancer and invoked the signaling mechanism by which TrkB.T1 sequesters GDI leading to activation of RhoA signaling (Li et al., 2009). In another study, a similar mechanism was proposed to cause morphological changes in C6 rat glioma cells (Ohira et al., 2006). More recently, the Holland lab experimentally tested *in vivo* whether expression of TrkB.T1 plays a role in gliomas. This study stems from the observation that TrkB.T1 is the predominant TrkB isoform expressed across a range of human gliomas and, surprisingly, that high transcript expression of TrkB.FL is associated with better, not worse, prognosis for both glioblastoma multiforme (GBM) and low grade gliomas (LGG; Pattwell et al., 2020). Using the elegant experimental paradigm that employs the RCAS-tv/a technology, they demonstrated that TrkB.T1 enhances PDGFB-driven tumors in mice, and the perdurance of PI3K and STAT3 signaling pathways (Pattwell et al., 2020). Together, these results demonstrate a previously unidentified role for TrkB.T1 in gliomas although how TrkB.T1 influences PI3K and STAT3 signaling is still unclear.

### Relevance of TrkB.T1 in Neurological Disorders

Diverse and region-specific dysregulation of BDNF-TrkB signaling has been observed in many neuropsychiatric disorders such as Alzheimer’s disease (AD), Parkinson’s disease, Amyotrophic Lateral Sclerosis (ALS), Huntington’s disease, epilepsy, stroke, mood disorders and schizophrenia. The consistent identification over the years of changes in the expression of truncated TrkB isoforms in these diseases has suggested that these isoforms are important transducers and determinants of dysfunctional BDNF signaling and possibly a critical causative factor underlying neuronal damage.

#### Amyotrophic Lateral Sclerosis

Amyotrophic Lateral Sclerosis is caused by rapidly progressing degeneration of the upper and lower motor neurons leading to muscle paralysis and death generally within 5 years of onset (Mitchell and Borasio, 2007). Two FDA approved agents, riluzole and edaravone, provide only a small benefit and are not curative. Lack of neurotrophic support is believed to be one of the causative factors leading to motor neuron loss (Bruijn et al., 2004). The initial findings that BDNF rescues injury-induced motor neurons death and motor dysfunction in the wobbler mouse model of motor neuron disease (Sendtner et al., 1992; Yan et al., 1992; Ikeda et al., 1995) provided the rationale for the clinical use of BDNF in ALS patients. However, in all clinical trials BDNF has failed to show any meaningful therapeutic effect (Group, 1999; Ochs et al., 2000; Kalra et al., 2003; Beck et al., 2005). While the lack of efficacy of BDNF has been attributed to its poor pharmacokinetics and pharmacodynamics properties, a number of observations in both human as well as rodent models of ALS have suggested that one of the causes of the underlying pathology in ALS is not the lack of BDNF supply but rather a defect in downstream TrkB signaling. Indeed, muscle of ALS patients has increased levels of BDNF (Kust et al., 2002). Moreover, total TrkB levels were either increased or unaltered in the postmortem analysis, yet there was a decrease in TrkB phosphorylation (Seeburger et al., 1993; Kawamoto et al., 1998; Mutoh et al., 2000). Similar results, i.e., increased BDNF levels associated with decreased TrkB phosphorylation, were observed in the plantaris muscle in the SOD1 G93A mutant mouse model of ALS (Just-Borras et al., 2019).

Based on these findings and the facts that in lumbar spinal cord of WT as well as SOD1 G93A mice, the levels of truncated TrkB.T1 receptors increase with age while that of TrkB.FL decreases we hypothesized that the presence of TrkB.T1 limits the pro-survival functions of BDNF -TrkB.FL signaling (Yanpallewar et al., 2012). This suggestion was supported by experiments in which TrkB.T1 deletion in mutant SOD1 mice delays the onset of the disease, slows down the motoneuron loss and improves mobility test results at the end stage of the disease compared with normal mutant SOD1 mice. Although, the increase in TrkB.T1 could be secondary to the increased astrogliosis in mutant spinal cord (cell types expressing only TrkB.T1) the findings that TrkB.T1 deletion does not change the SOD1 spinal cord inflammatory state suggests that this receptor does not influence microglia or astrocyte activation (Zhang and Huang, 2006; Yanpallewar et al., 2012, 2021; Just-Borras et al., 2019). These observations strongly suggest a role for TrkB.T1 in the pathophysiology of ALS and alternative ways to activate TrkB.FL may be needed to overcome the insufficient or defective TrkB signaling.

#### Alzheimer’s Disease

Alzheimer’s Disease is the most common type of dementia (Querfurth and LaFerla, 2010) with cognitive impairments associated with loss of cholinergic neurons and formation of plaques due to the deposition of beta-amyloid and neurofibrillary tangles formed by hyper-phosphorylated tau protein. Because loss of cholinergic neurons is a major feature of AD it was suggested that diminished NGF-mediated neurotrophic support is a major cause of disease. However, there is also strong evidence that dysregulation of BDNF-TrkB signaling can be implicated in AD. For example, BDNF mRNA and protein levels as well as protein levels for the TrkB.FL isoform have been found to be reduced in postmortem brain samples of AD patients, while TrkB.T1 is increased (Phillips et al., 1991; Ferrer et al., 1999). Moreover, in a separate study of AD brains, a specific increase in the truncated TrkB.Shc isoform has been reported in the hippocampus. This isoform, found mainly in humans and not in mouse, can, like TrkB.T1, inhibit TrkB.FL function (Wong et al., 2012). Although the mechanistic role of TrkB receptors isoform dysregulation in AD is still largely unknown, in neuronal cultures, amyloid beta has been found to increase TrkB.T1 levels while decreasing TrkB.FL. Parallel to this finding, TrkB.T1 overexpression in the APPswe/PS1dE9 transgenic mouse model of AD exacerbated spatial learning and memory impairment whereas overexpression of TrkB.FL improved it (Kemppainen et al., 2012). Importantly, the relevance of BDNF/TrkB signaling in AD has been further validated by the findings that BDNF exerts neuroprotective effects in rodent and primate models of AD (Nagahara et al., 2009).

#### Stroke and Neuronal Trauma

Stroke is the leading cause of disability and death in developed countries. Ischemic (caused by blockade of a blood vessel) or hemorrhagic (caused by a ruptured vessel) stroke leads to reduced supply of oxygen and nutrients ultimately resulting in neuronal death. In ischemic stroke, levels of BDNF are decreased in the infarct core while there is a rapid and sustained upregulation in the peri-infarct area. This increase in BDNF is believed to be a compensatory mechanism to provide trophic support to neurons (Lindvall et al., 1992, 1994, 1996). Analysis of TrkB expression in human stroke necropsy samples and in animal models of ischemia shows a significant up-regulation of TrkB.T1 that appears neuro-specific, while the levels of TrkB.FL decreases (Vidaurre et al., 2012). One of the mechanisms contributing to the altered TrkB isoforms expression appears related to alternative mRNAs splicing favoring the expression of TrkB.T1 over TrkB.FL. A second mechanism appears related to the calpain-dependent degradation of TrkB.FL. While it is unclear why there is a change in the splicing of TrkB isoforms, it is possible that an alteration in the expression of specific RNA binding proteins caused by the excitotoxic insult could lead to such dysregulation (Tomassoni-Ardori et al., 2019). The *in vitro* rescue of neurons from excitotoxic death by restoration of the TrkB-FL/TrkB-T1 balance suggests that dysregulation of TrkB isoform expression may be one of the causes leading to neuronal cell-death during stroke (Vidaurre et al., 2012; Tejeda and Diaz-Guerra, 2017). A better understanding of this pathophysiological mechanism may lead to improved therapies for this brain injury.

Truncated TrkB.T1 levels are also increased following traumatic brain (Rostami et al., 2014) and spinal cord injury in the area surrounding the damaged tissues, suggesting a role in limiting neurotrophin availability at the lesion site (King et al., 2000; Wu et al., 2013). Up-regulation of TrkB.T1 mRNA is not just limited to the acute phase of the damage but is sustained up to 8 weeks in both a rat model of penetrating traumatic brain injury as well as a mouse model of spinal cord contusion injury (SCI; Rostami et al., 2014; Matyas et al., 2017). Moreover, deletion of TrkB.T1 in astrocytes not only reduces inflammatory response but also improves impaired motor function and neuropathic pain after SCI (Matyas et al., 2017). Curiously, microarray analysis of spinal cord tissue after injury in WT and TrkB.T1 KO mice has also found a differential regulation of cell cycle associated genes although the significance of this changes is still unclear (Wu et al., 2013; Wei et al., 2019). Further studies are needed to investigate if modulating TrkB.T1 expression can indeed influence the recovery after traumatic injuries of the nervous system and whether it can be targeted to develop treatments to improve the outcome of trauma in patients.

#### Parkinson’s Disease

Abundant data have suggested a role for impaired BDNF/TrkB signaling in the etiology of Parkinson’s disease, at least from *in vivo* animal models. For example, ablation of the BDNF gene impairs the survival and/or maturation of substantia nigra (SN) dopamine (DA) neurons during development (Baquet et al., 2005); loss of one copy of TrkB leads to an age-dependent increase in the levels of α-synuclein in the SN (von Bohlen und Halbach et al., 2005), and in a mouse model with a chronic reduction in TrkB signaling (∼30% of WT) there is an age-dependent and selective degeneration of SN DA neurons and increased vulnerability of these neurons to neurotoxins (Baydyuk and Xu, 2014). Because it has also been reported that neurons of PD patients have an increase in TrkB.T1 expression (Fenner et al., 2014) and neurons derived from iPSCs of PD patients have elevated RBFOX1 (Lin et al., 2016) it will be of interest to investigate whether TrkB.T1 upregulation in PD is among the determinants of this pathology.

#### Schizophrenia

Patients with schizophrenia show cognitive and perception deficits that are linked to abnormalities in the function of the dorsolateral prefrontal cortex. Interestingly, in this brain region of deceased schizophrenic patients, BDNF and TrkB.FL are significantly decreased, while TrkB.Shc and TrkB.T1 isoforms are upregulated (Durany et al., 2001; Weickert et al., 2003, 2005; Hashimoto et al., 2005; Wong et al., 2013). These findings have, once again, suggested an imbalance in neurotrophic BDNF signaling in these patients (Yuan et al., 2010). The mechanism underlying dysregulation of TrkB isoform receptor is unknown but the recent report of alternative splicing characteristics of a growing number of schizophrenia risk genes suggests that the changes in TrkB splicing may be part of wider genome alterations in the splicing associated with schizophrenia pathogenesis (Zhang et al., 2021).

#### Stress Disorders

A role for BDNF-signaling in the pathophysiology of stress disorders such as depression has been extensively studied. Stress, one of the major risk factors underlying mood disorders, decreases BDNF and its downstream signaling whereas antidepressant therapies exert their therapeutic effect, at least in part, through promotion of this signaling pathway (Yang et al., 2020). Specifically, there is altered expression of TrkB.T1 in postmortem brains of suicide victims (Dwivedi et al., 2003; Ernst et al., 2009). Importantly, the observed TrkB isoform imbalance is believed to be due to microRNA Hsa-miR-185* mediated modulation of TrkB.T1 levels (Maussion et al., 2012).

Taken together, these data strongly implicate altered expression of truncated TrkB isoforms in many neurological disorders. Validation of these findings in more animal models should lead to a better understanding of whether an imbalance in Trk receptor isoform expression is a major factor underlying the pathogenesis of these neural diseases.

## Therapeutic Relevance and Future Perspective

Dysregulation of homeostatic BDNF signaling has been associated with a number of neuropsychiatric disorders. Most therapeutic strategies aimed at restoring or enhancing BDNF “trophic” support have focused on exogenous administration of BDNF (e.g., in ALS) and the development of BDNF agonists binding to the TrkB extracellular domain such as TrkB activating antibodies (Lin et al., 2008; Perreault et al., 2013; Guo et al., 2019). To date, the search for small molecule agonists has been mainly unsuccessful, or questionable at best (Todd et al., 2014; Boltaev et al., 2017; Pankiewicz et al., 2021). The delivery of BDNF has universally failed as a therapeutic agent probably due to its inability to cross the BBB, the short half-life, and the difficulties of delivery to specific brain areas in a precise spatio-temporal fashion (Miranda-Lourenco et al., 2020). A variety of approaches are currently being pursued to address the issue of BDNF delivery, including the use of biomaterials and nanoparticles, viral mediated gene delivery and transplantation of neurotrophin-producing cells (Houlton et al., 2019; Jarrin et al., 2021). Unfortunately, most of these approaches ignore the possibility that changes at the level of TrkB receptor, including TrkB.T1 upregulation, may influence the downstream BDNF signaling and therefore the therapeutic outcome. Indeed, upregulation of TrkB.T1 in a trisomic mouse model renders hippocampal neurons completely unresponsive to BDNF both *in vitro* and *in vivo*. Importantly, restoration of TrkB.T1 to physiological levels rescues neurons sensitivity to BDNF and neuronal cell death *in vivo* (Dorsey et al., 2002, 2006). These observations have critical implications for the therapeutic use of TrkB agonist antibodies that bind selectively to TrkB. While these antibodies do not engage p75NTR and have a longer half-life (days instead of minutes to hours seen with BDNF) allowing for better diffusion into the neural tissues (Guo et al., 2019; Han et al., 2019), they still bind to TrkB.T1 with the risk of being sequestered and neutralized. The data presented in this review therefore warrants a critical reexamination of these approaches because neurons may not suffer from a deficit of neurotrophin supply but rather an intrinsic block of signaling at the receptor level. Interestingly, transactivation of TrkB receptors seems to circumvent this issue. Use of adenosine A2A receptor agonists that transactivate TrkB intracellularly have been shown to enhance survival of lesioned facial motoneurons (Wiese et al., 2007) and lead to a delay in disease progression in a mouse model of ALS (Domeniconi and Chao, 2010; Yanpallewar et al., 2012). A similar transactivation of TrkB by glucocorticoids has been shown to promote downstream signaling and exert neurotrophic effect (Jeanneteau et al., 2008). Since, the TrkB transactivation mechanism occurs independently of BDNF and is not influenced by the presence of TrkB.T1 at the membrane, it is possible to increase the ability of diseased neurons to respond to TrkB signaling. One caveat is that TrkB.T1, as described earlier, can independently affect calcium signaling and imbalances in this isoform levels may influence neuronal survival by altering intracellular calcium levels. Indeed, dysregulation of calcium homeostasis in spinal motoneurons of SOD1 mutant mice has already been reported (Damiano et al., 2006; Guatteo et al., 2007). This scenario calls for the consideration of multiple strategies to identify the therapeutic potential of BDNF-TrkB signaling: one to reduce TrkB.T1 to correct its dominant-negative function and neuronal calcium levels and the other to increase activation of TrkB.FL by transactivation. An attractive approach to regulate aberrant TrkB isoforms expression is by targeting TrkB alternative splicing (AS). By targeting AS it is possible to regulate the alternate exon inclusion or block exon skipping to enhance read-through of full length isoform or even blocking a splice site to promote formation of a particular splice variant (Graziewicz et al., 2008; Geib and Hertel, 2009; Lin et al., 2015). This approach has now become reality as a therapeutic antisense oligonucleotide (ASO) that binds to the survival motor neuron 2 (SMN2) messenger RNA has been approved by the FDA for the treatment of spinal muscular atrophy (SMA). Treatment with the steric block ASO Nusinersen, that binds to the SMN2 mRNA, was found to promote exon 7 inclusion resulting in increased full length SMN protein. This ultimately leads to the increased survival and motor function in patients of SMA (Wood et al., 2017). A similar approach could be used to correct TrkB.T1 upregulation since even limited efficacy in correcting aberrant splicing levels of TrkB.T1 may lead to significant therapeutic benefits and may even be more desirable considering that TrkB.T1 is important for the normal function of other organs such as the heart and the endocrine pancreas.

## Data Availability Statement

The original contributions presented in the study are included in the article/supplementary material, further inquiries can be directed to the corresponding author.

## Author Contributions

LT and SY wrote the manuscript. Both authors contributed to the article and approved the submitted version.

## Conflict of Interest

The authors declare that the research was conducted in the absence of any commercial or financial relationships that could be construed as a potential conflict of interest.

## Publisher’s Note

All claims expressed in this article are solely those of the authors and do not necessarily represent those of their affiliated organizations, or those of the publisher, the editors and the reviewers. Any product that may be evaluated in this article, or claim that may be made by its manufacturer, is not guaranteed or endorsed by the publisher.
